# Characterization of *Thermotoga neapolitana* Alcohol Dehydrogenases in the Ethanol Fermentation Pathway

**DOI:** 10.3390/biology11091318

**Published:** 2022-09-05

**Authors:** Chong Sha, Qiang Wang, Hongcheng Wang, Yilan Duan, Chongmao Xu, Lian Wu, Kesen Ma, Weilan Shao, Yu Jiang

**Affiliations:** 1 Biofuels Institute, School of the Environment, Jiangsu University, Zhenjiang 212013, China; 2 School of Biological and Food Engineering, Suzhou University, Bianhe Middle Road 49, Suzhou 234000, China; 3 Huzhou Research Center of Industrial Biotechnology, Shanghai Institutes for Biological Sciences, Chinese Academy of Sciences, Huzhou 313000, China; 4 Department of Biology, University of Waterloo, Waterloo, ON N2L 3G1, Canada; 5 Shine E BioTech (Nanjing) Company, Nanjing 210023, China

**Keywords:** cellulosic ethanol, *Thermotoga* spp., hyperthermophilic enzyme, alcohol dehydrogenase, acetaldehyde dehydrogenase

## Abstract

**Simple Summary:**

Hyperthermophilic *Thermotoga* spp. are promising candidates for the metabolic engineering of cellulosic ethanol producing strains. However, *Thermotoga* spp. are hydrogen producers, and none of them have been found to produce significant amount of ethanol. In this study, we focused on the enzymes responsible for the part of the ethanol production pathway from ac-CoA to ethanol. Aldehyde dehydrogenases (Aldhs) and alcohol dehydrogenases (Adhs) are the vital enzymes involved in the metabolism of ethanol in *Thermotoga* spp.. However, the biochemical properties of a number of Adhs related to ethanol formation and consumption have not been identified until now. The aim of this study was to determine the role of putative Adhs in *T. neapolitana* (*Tne*) in the pathway from ac-CoA to ethanol. We found that the Fe-AAdh (encoded by CTN_0580) and Fe-Adh2 (encoded by CTN_1756) were the main enzymes responsible for ethanol production in *Tne*. While Zn-Adh (encoded by CTN_0257) was identified as the main protein responsible for ethanol consumption in the acid environment and Fe-Adh1 (encoded by CTN_1655) played a minor role in ethonal production and consumption in *Tne*. Basing on these results, it will be helpful to construct and assemble a novel hyperthermophilic strain to produce cellulosic ethanol.

**Abstract:**

Hyperthermophilic *Thermotoga* spp. are candidates for cellulosic ethanol fermentation. A bifunctional iron-acetaldehyde/alcohol dehydrogenase (Fe-AAdh) has been revealed to catalyze the acetyl-CoA (Ac-CoA) reduction to form ethanol via an acetaldehyde intermediate in *Thermotoga neapolitana* (*T. neapolitana*). In this organism, there are three additional alcohol dehydrogenases, Zn-Adh, Fe-Adh1, and Fe-Adh2, encoded by genes CTN_0257, CTN_1655, and CTN_1756, respectively. This paper reports the properties and functions of these enzymes in the fermentation pathway from Ac-CoA to ethanol. It was determined that Zn-Adh only exhibited activity when oxidizing ethanol to acetaldehyde, and no detectable activity for the reaction from acetaldehyde to ethanol. Fe-Adh1 had specific activities of approximately 0.7 and 0.4 U/mg for the forward and reverse reactions between acetaldehyde and ethanol at a pH_opt_ of 8.5 and T_opt_ of 95 °C. Catalyzing the reduction of acetaldehyde to produce ethanol, Fe-Adh2 exhibited the highest activity of approximately 3 U/mg at a pH_opt_ of 7.0 and T_opt_ of 85 °C, which were close to the optimal growth conditions. These results indicate that Fe-Adh2 and Zn-Adh are the main enzymes that catalyze ethanol formation and consumption in the hyperthermophilic bacterium, respectively.

## 1. Introduction

Biomass (lignocellulose) is widespread in nature, and has been in the limelight for second-generation bioethanol production, a promising alternative to fossil fuels. Therefore, it is not surprising that the metabolism and bioconversion of biomass attracts much attention [1,2]. *Escherichia coli*, *Saccharomyces cerevisiae*, and *Zymomonas mobilis* are considered as the main ethanol-producing strains because a high ethanol concentration and yield can be achieved via metabolic engineering [3,4,5]. *Z. mobilis* is one of the most typical strains for two-step ethanol fermentation for its high ethanol productivity, ethanol tolerance, and genetic manipulation feasibility [5]. The two-step pathway is catalyzed by pyruvate decarboxylase (PDC) and alcohol dehydrogenase (ADH). A synthetic metabolic pathway, based on the heterologous genes encoding levoglucosan kinase, PDC and Adh, was constructed and introduced into *E. coli* to improve ethanol production [4]. A maximum ethanol concentration of 23.8% was also obtained from starch biomass by *S. cerevisiae* without engineering [6]. However, these strains cannot use complex substrates (such as wood and straw) and lignocellulose usually cannot be served as a microbial substrate directly unless depolymerized into fermentable sugars [2,7,8]. On the other hand, wild-type *S. cerevisiae* and *Z. mobilis* cannot usually metabolize pentose sugars unless they are engineered properly. All of these limitations, including the requirement of a complex pretreatment and co-expression of heterologous genes in the target strains, will impede bioethanol production.

Thermophilic anaerobic bacteria have received increasing interest as a promising bioprocessing platform for bioethanol production due to their broad substrate range and higher operating temperature, like bacteria within the genera of *Thermoanaerobacterium*, *Thermoanaerobacter*, and *Clostridium*, which have good ethanol yields [9,10,11,12]. The ethanol production of *C. thermocellum* was enhanced significantly by introducing *adh*A, *adh*E, and *nfn*AB simultaneously from *T. saccharolyticum* [13]. The final concentration of ethanol reached 45.01 g/L by the mutant strain *Thermoanaerobacterium aotearoense* SCUT27/Δldh/ΔpflA, which was grown anaerobically at an initial pH of 6.5 and 55 °C [14].

Hyperthermophilic anaerobic bacteria (optimal growth temperature ≥80 °C) are considered to be one of the most promising candidates for the production of bioethanol from complex lignocellulosic biomass, with great advantages of a broad substrate range, higher operating temperatures, higher ethanol diffusion rates, and lower cooling and distillation costs, as well as a lower risk of microbial contamination [14,15]. Unit operations and processes have been developed for the pretreatment of biomass, enzymatic hydrolysis of the cellulosic component, co-fermentation of pentose and hexose sugars for cellulosic ethanol production, and ethanol distillation. How to integrate these units for optimization at system levels presents another challenge [16]. *Thermotoga* spp. could secrete a series of thermostable cellulases and hemi-cellulases, and could also grow at 80 °C (near the ethanol distillation temperature), which make *Thermotoga* spp. more suitable for integrating these units into consolidated bioprocessing in one pot and potentially reducing the operating cost. Cooling is considered to be a significant cost of the industrial fermentation process (mesophilic), so this may not be needed for the process at high temperatures, at which, the separation of bioethanol may be carried out in situ [17,18,19]. *Thermotoga* spp. are also cellulolytic and hemicellulolytic hyperthermophiles that meet the requirements for the construction of a cellulosic biofuel producer. Hydrogen production by *Thermotoga* spp. was tested using soluble starch or CMC or cellulose as the carbon source. The strains showed a 39–49 mL H_2_/L culture from cellulose, 172–175 mL H_2_/L culture from CMC, and 345–404 mL H_2_/L culture from soluble starch [20].

Researchers have been focusing their efforts on aldehyde dehydrogenase (Aldh) and Adh since 1988 [21,22]. The bacterial alcohol dehydrogenase (AdhA) was inserted into the archaeon *Pyrococcus furiosus* and the engineered stain produced more than 20 mM ethanol via acetate and acetaldehyde [23]. The pyruvate ferredoxin oxidoreductases (POR or PFOR) from *T. maritima* and *T. hypogea* were found to have CoA-dependent PDC activity, where the PDC activity is less than 2% of POR activity [24,25,26]. Thus, more than 98% of pyruvate is probably converted to acetyl-coenzyme A (Ac-CoA) by POR, and ac-CoA is further converted to acetic acid if there is no enzyme to catalyze the reduction of ac-CoA to produce acetaldehyde. Coincidentally, a putative iron-containing alcohol dehydrogenase (Fe-AAdh encoded by CTN_0580) was firstly revealed to be a bifunctional aldehyde/alcohol dehydrogenase that catalyzed both reactions from ac-CoA to acetaldehyde, and from acetaldehyde to ethanol [27].

It is true that some alcohol dehydrogenases from *Thermotoga* spp. were characterized and the crystal structure was obtained [28]. However, there is a lack of understanding of the physiological roles of all three alcohol dehydrogenases involved in the ethanol metabolism in *Thermotoga* spp. In this study, we focused on the enzymes responsible for the part of the ethanol production pathway from ac-CoA to ethanol. Aldehyde dehydrogenases (Aldhs) and alcohol dehydrogenases (Adhs) are the vital enzymes involved in the metabolism of ethanol in *Thermotoga* spp. However, the biochemical properties of a number of Adhs related to ethanol formation and consumption have not been identified until now. The aim of this study was to determine the role of putative Adhs in *T. neapolitana* in the pathway from ac-CoA to ethanol.

## 2. Materials and Methods

### 2.1. Bacterial Strains and Plasmids

*E. coli* JM109 (Promega) was used as host for cloning and expression of genes in a pHsh vector constructed by this laboratory (Shine E Biotech, Nanjing, China). *E. coli* cells were routinely grown aerobically in Luria–Bertani (LB) medium at 30 °C, and 100 μg/mL ampicillin was added to the LB medium for selective cultures. The plasmids pHsh-1655, pHsh-1756, and pHsh-0257 were constructed previously [27].

### 2.2. Gene Expression and Enzyme Purification

*E. coli* JM109 cells were transformed using the expression plasmids containing the Adh genes and were grown at 30 °C to an OD_600_ of 0.6–0.8 before the cultures were transferred to a 42 °C shaking water-bath incubator for heat-shock induction of gene expression. After 6 h of cultivation along with gene expression at 42 °C, cells were harvested and transferred into an anaerobic chamber for the isolation and purification of recombinant enzymes. *E. coli* cells were resuspended in degassed 3-(N-morpholino) propanesulfonic acid buffer (MOPS, 25 mM, pH 6.5) and disrupted by sonication, followed by heat treatment at 75 °C for 30 min.

After the cell debris and heat-denatured protein were removed by centrifugation (14,000× *g*, 30 min), the supernatants were mixed with the same volume of 2× binding buffer and loaded to the Ni-metal affinity column (Novagen) according to the manufacturer’s instructions. Each recombinant enzyme bound to Ni-column was eluted and collected into a dialysis bag, concentrated by embedding the dialysis bag in PEG 20,000, and dialyzed against MOPS with two changes. Purified enzymes were stored in MOPS with the addition of 10% glycerol, 1 mM dithiothreitol (DTT), 0.02% (*w*/*v*) NaN_3_, and 1 mM FeCl_2_ or ZnCl_2_ for Fe-Adhs or Zn-Adh, respectively.

Protein expression levels and the purity of recombinant proteins were examined by SDS-PAGE [29]. Protein concentration was determined by measuring the absorbance at 280 nm and calculating the concentration based on the extinction coefficient for the amino acid sequence of each enzyme (the extinction coefficient was estimated by the online tool ProtParam: https://www.expasy.org/resources/protparam, accessed on 6 April 2021).

### 2.3. Enzyme Activity Assay

Adh activities were measured by monitoring the changes in NAD(P)H absorbance at 340 nm. One unit of enzyme activity was defined as the amount of enzyme producing or consuming 1 μmol of NAD(P)H per min. All of the activity assays were performed anaerobically in triplicate, and data reported here are the mean of the three replicates. For standard enzyme activity assays, the reaction mixture consisted of 100 μL of MOPS containing suitable substrates at concentrations: 1 mM NAD(P)^+^ or NAD(P)H, and 20 mM acetaldehyde or ethanol, at different pH (4–10) and temperatures (50–100 °C) as indicated for each reaction.

## 3. Results

### 3.1. Gene Expression and Enzyme Purification

The plasmids pHsh-1655, pHsh-1756, and pHsh-0257 were constructed previously [27]. The recombinant proteins produced from *T. neapolitana* genes CTN_1655, CTN_1756, and CTN_0257 were designated as Fe-Adh1, Fe-Adh2, and Zn-Adh, respectively. These enzymes were purified to near gel electrophoresis homogeneity after heat treatment and metal affinity chromatography using a Ni-column at room temperature. However, the purified Fe-Adh1 and Fe-Adh2 became inactivated within approximately one week after they were stored at −20 °C, implying that they were sensitive to O_2_ in the air and buffer. This problem was resolved by purifying these enzymes in an anaerobic chamber using degassed buffers and keeping the purified enzyme in sealed anaerobic serum tubes.

### 3.2. Biochemical Properties of the Recombinant Enzymes

**Figure 1:**
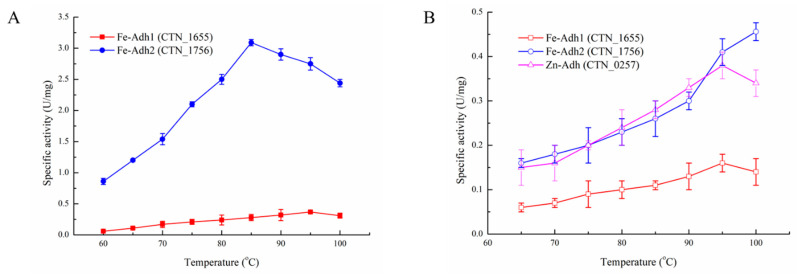
The effects of temperatures on the enzymatic activity of the three Adhs from *T. neapolitana*: (**A**) forward reaction for Fe-Adh1 and Fe Adh2; (**B**) reverse reaction for Fe-Adh1, Fe-Adh2, and Zn-Adh. All of the enzymatic activity was determined at pH 7.0. All of the activity assays were performed in triplicate, and data reported here are the mean of the three replicates.

**Figure 2:**
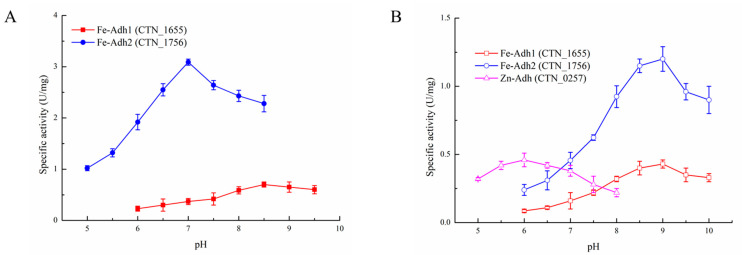
The effects of pH on the enzymatic activity of the three Adhs from *T. neapolitana*: (**A**) forward reaction for Fe-Adh1 and Fe Adh2; (**B**) reverse reaction for Fe-Adh1, Fe-Adh2, and Zn-Adh. All of the enzymatic activity was determined at the optimal temperature. All of the activity assays were performed in triplicate, and data reported here are the mean of the three replicates.

The biochemical properties of enzymes were first characterized at different reaction pH and temperatures. Fe-Adh1, Fe-Adh2, and Zn-Adh did not show any ac-CoA reduction activity [27]. However, for the forward reaction in the last step of this pathway, Fe-Adh2 could catalyze acetaldehyde to produce ethanol at 85 °C and pH 7.0, while Fe-Adh1 reached the highest enzymatic activity at a higher temperature 95 °C and pH 8.5 (Figure 1A and Figure 2A). Zn-Adh did not show any detectable activity for the forward reaction. For the reverse reaction (from ethanol to ac-ald), Fe-Adh1 and Zn-Adh reached the highest activity at 95 °C, while the optimal temperature is more than 100 °C for Fe-Adh2 (Figure 1B). Further, Fe-Adh1 and Fe-Adh2 had a similar trend for the pH dependency, and the reverse reaction occurred optimally in a more alkaline environment at pH 9.0. Conversely, Zn-Adh had a much lower optimal pH of 6.0, which is near the physiological value (Figure 2B).

### 3.3. Cofactor Dependence for the Three Putative Adhs

The cofactor dependence of these enzymes was characterized at their optimal pH and temperatures in each step (Table 1). When analyzed at 85 °C and pH 7.0, the activity of Fe-Adh2 to catalyze ac-ald reduction only depended on the cofactor NADPH, and its specific activity was up to 3.09 U/mg, which was much higher than that of Fe-Adh1 (Table 1). For the reverse reaction (ethanol oxidation), compared to NAD^+^, NADP^+^ was preferably served as the cofactor by the three Adhs. Although the specific activity of Zn-Adh was similar to that of Fe-Adh1 and much lower than that of Fe-Adh2, the reverse reaction would probably be driven by Zn-Adh due to the near physiological pH.

### 3.4. Involvement in the Ethanol Metabolic Pathway in T. neapolitana

The last two steps of ethanol fermentation in anaerobes are two reversible redox reactions, from ac-CoA to acetaldehyde catalyzed by the CoA-dependent aldehyde dehydrogenase (Aldh), and from acetaldehyde to ethanol catalyzed by Adh. Fe-AAdh was the first enzyme found in hyperthermophiles that catalyzed the reduction of ac-CoA to produce acetaldehyde [27]. Fe-Adh1 and Fe-Adh2 catalyzed the forward and reverse reactions between acetaldehyde and ethanol without showing any ac-CoA reduction activity. However, the activity of Fe-Adh1 was much lower than that of Fe-Adh2. In comparison, Fe-Adh1 not only had lower activities, but also had higher pH and temperature optima than Fe-Adh2. The *T. neapolitana* Zn-Adh specifically catalyzed the reaction from ethanol to acetaldehyde, and no activity was detectable for the reaction from acetaldehyde to ethanol. This result is different from almost all of the other Adhs reported previously [21,22,30]. The activities of these enzymes depended highly on the pH conditions of the reaction mixtures. *T. neapolitana* Zn-Adh reacted optimally at pH 6.0, which was close to pH 6.5, the pH value of non-buffered cell-free extracts of *T. neapolitana*. The CoA-independent Aldh (encoded by CTN_1548) had an optimal pH of 9.5 (data not shown), which, together with Zn-Adh, could possibly form a detoxification system for the oxidation of ethanol and aldehyde.

## 4. Discussion

Ethanol is an important renewable fuel that could be produced from pyruvate via PDC, or from ac-CoA via bifunctional aldehyde/alcohol dehydrogenase (such as AdhE) [31]. At present, research has been focused on the identification of thermophilic enzymes involved in the ethanol fermentation pathway [32]. The pyruvate ferredoxin oxidoreductases (POR or PFOR) from *T. maritima* and *T. hypogea* were found to convert 98% of pyruvate to ac-CoA [24,26]. The CoA-dependent Aldh activity is performed by the enzymes annotated as alcohol dehydrogenases, such as AdhB and AdhE, in anaerobic thermophiles [11,33,34,35].

Four possible reactions could occur in the final steps of ethanol formation from ac-CoA. To determine the number of steps each dehydrogenase can catalyze, the biochemical properties of the enzymes in *T. neapolitana* were characterized at their optimal reaction pH and temperatures (Table 1). When searching for an enzyme that could reduce ac-CoA to acetaldehyde, we found that the purified enzyme encoded by CNT_0580 was a bifunctional aldehyde/alcohol dehydrogenase (Fe-AAdh) that could catalyze all of the forward and reverse reactions in the pathway, from ac-CoA to ethanol, under optimal reaction conditions [27]. As shown in Figure 1 and Figure 2, we identified the biochemical properties of the other three Adhs, and concluded that Fe-Adh2 and Fe-AAdh were the main reactive enzymes for ethanol formation and that Zn-Adh could probably form a detoxification system for the oxidation of ethanol together with Aldh (encoded by CTN_1548). However, only Fe-AAdh had a weak ac-CoA reduction activity, indicating that ac-CoA reduction was the rate-limiting step for ethanol formation in *T. neapolitana*.

**Figure 3:**
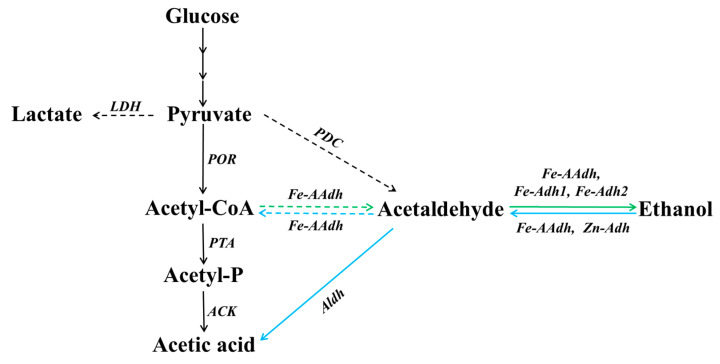
Depicted is the end of ethanol metabolic pathway in *T. neapolitana*. The dotted line is a weak reaction; the green arrows are the forward reaction (ethanol formation); the blue arrows represent the detoxifying path. LDH: lactate dehydrogenase; POR: pyruvate: ferredoxin oxidoreductase; PDC: pyruvate decarboxylase; PTA: phosphate acetyltransferase; ACK: acetate kinase; Aldh: aldehyde dehydrogenase; Fe-AAdh: bifunctional iron-acetaldehyde/alcohol dehydrogenase; Fe-Adh1, Fe-Adh2, and Zn-Adh: Fe-/Zn- alcohol dehydrogenase.

Therefore, Fe-AAdh and Fe-Adh2 were the main reactive enzymes for ethanol formation at the pH range that was approximately the intracellular pH. The biochemical properties imply that these enzymes contribute differently in ethanol metabolism, and some activities may be very low in a living cell, where the pH value, temperature, and substrate concentration are usually limited to certain levels. Based on these results, we propose the involvement of these enzymes in the ethanol metabolic pathway from ac-CoA to ethanol via acetaldehyde as intermediate in *T. neapolitana* (Figure 3).

Hyperthermophilic *Thermotoga* spp. are promising candidates for the metabolic engineering of cellulosic ethanol-producing strains. However, *Thermotoga* spp. are hydrogen producers, and none of them have been found to produce significant amounts of ethanol. The reduction of ac-CoA is the first step in dumping the reducing power in the ethanol fermentation pathway, and the activity of Fe-AAdh and Fe-Adh2 may need to be triggered by high concentrations of the reduced nicotinamide cofactors (NADH and NADPH). However, there is strong Fe-hydrogenase to utilize ferredoxin and NADH synergistically for anaerobic H_2_ production so that H_2_ becomes the main fermentation product of *Thermotoga* spp. [36].

The major fermentation non-gaseous end-products of *Thermotoga* spp. include acetate, lactate, and ethanol. Metabolic engineering has proven to be an effective approach to constructing novel ethanol-producing strains. By the heterologous expression of a thermostable acetyl-CoA synthetase that catalyzed the irreversible acetate assimilation in *T. neapolitana*, a strongly increased lactate production (up to 2.5 g/L) was achieved by the recombinant stain from acetate and CO_2_ [37]. These engineering attempts could be grouped into two categories: the first one focused on eliminating native competing metabolic pathways, such as organic acid production [38,39] or hydrogen production [40]. The second one focused on introducing heterologous genes that are a part of well-characterized ethanol-producing pathways into hyperthermophiles [41,42]. For example, by the heterologous co-expression of *adhA* and *adhE* from *Thermoanaerobacter*, the highest amount of ethanol (estimated 61% theoretical yield) was produced in *P. furiosus* [43]. The insertion of a single gene encoding the thermostable NADPH-dependent AdhA (Tte_0696) from *Caldanaerobacter subterraneus* resulted in a high ratio of ethanol over acetate (>8:1) and enabled ethanol production up to 85 °C, the highest temperature for bio-ethanol production reported to date [44].

The cellulosic ethanol fermentation at temperatures above the ethanol boiling point has advantages in reducing the costs for autoclaving raw-materials and distilling ethanol from fermentation, which also favors the consolidate process of biomass degradation, ethanol fermentation, and distillation [14,15]. By eliminating the pathways for H_2_ and lactate formation, improving the activity of bifunctional aldehyde/alcohol dehydrogenase (Fe-AAdh), or even strengthening the coenzyme (NADPH) cycles, it will be a novel strategy for the construction and assembling of a hyperthermophilic strain to produce cellulosic ethanol.

## 5. Conclusions

In this study, three putative Adh genes in the *T. neapolitana* genome were investigated for their roles in ethanol metabolism for the first time. We report that the Fe-AAdh (encoded by CTN_0580) and Fe-Adh2 (encoded by CTN_1756) were the main enzymes responsible for ethanol production in *T. neapolitana* while Zn-Adh (encoded by CTN_0257) was identified as the main protein responsible for ethanol consumption in the acid environment and Fe-Adh1 (encoded by CTN_1655) played a minor role in ethanol production and consumption in *T. neapolitana*.

## Figures and Tables

**Table 1:** Optimal reaction conditions and cofactor dependence for the Adhs of *T. neapolitana* ethanol fermentation pathway.

Enzyme (Gene)	Fe-Adh1 (CTN_1655)		Fe-Adh2 (CTN_1756)		Zn-Adh (CTN_0257)	
**Reaction**	Ac-ald→Eth	Ac-ald←Eth	Ac-ald→Eth	Ac-ald←Eth	Ac-ald→Eth	Ald←Eth
**pH_opt_**	8.5	9.0	7.0	9.0	ND	6.0
**T_opt_**	95 °C	95 °C	85 °C	100 °C	ND	95 °C
**Activity (U/mg)**						
**NADPH**	0.70 ± 0.05		3.09 ± 0.06		ND	
**NADH**	ND		ND		ND	
**NADP**		0.43 ± 0.05		1.20 ± 0.06		0.46 ± 0.03
**NAD**		0.22 ± 0.04		0.25 ± 0.04		0.30 ± 0.02

Abbreviations: Ac-CoA, acetyl-CoA; Ac-ald, acetaldehyde; Eth, ethanol; ND, activity not detectable.

## Data Availability

Data sharing is not applicable to this article.
